# 

**Published:** 1891-12-12

**Authors:** 


PRICE ONE PENNY.

No. 272, Vol. XI.—December 12th, 1891.

[Registered at the General Post Office as a Newspaper for transmission in the United Kingdom and Abroad.]

WITH EXTRA NURSING SUPPLEMENT.
Per Annum, 6s. 6d., post free.
Monthly Parts, 6d.; post free, 8d.
Ditto per Annum, 8s., post free.

### THE HOSPITAL

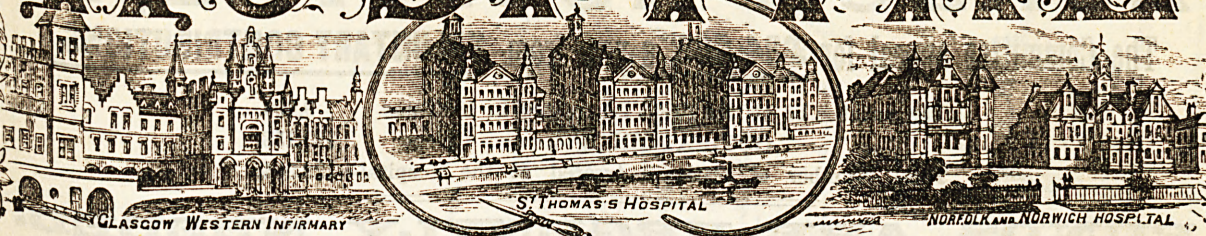

A WEEKLY JOURNAL OF

### Science, Medicine, Nursing, and Philanthropy.

SATURDAY, DECEMBER 12th, 1891.

**A Warning Outbreak** . . . . . . . . 121

**Passing Topics.**—How is it Done? . . . . . . . 122

**Some Scientific Aspects of Insanity.** VI.—Early Stages 123

**Letters from Far Japan.** By Mrs. Ernest Hart . . . 123

**Some American Hospitals.** I.—Introductory . . . 125

**Notes and News** . . . . . . . . . 126

**General Charities—Scraps and Gleanings** . . . . 127

**Annotations.**—Medicine and Surgery at Toronto—The Carpenters' Company and the Sanitary Institute . . . 128

**The Practitioner's Mirror and Retrospect—**

Medicine.—The Bacteriology of Diphtheria . . . . 129

Surgery.—"Antiseptic" and "Aseptic" Surgery . . 130

Gynæcology.—Dr. Oscar Bloch on Removal of Ovaries . 131

Ophthalmology.—Iritis . . . . . . . . . 131

New Drugs, Appliances, and Things Medical—At the British Medical Association (*concluded*) . . . . 132

**The Student of Medicine.**—Editorial Notes—Notes from the Schools—Appointments—Vacancies.

EXTRA SUPPLEMENT.—THE NURSING MIRROR.

**En Passant.** — Leper Children — Short Items — Belfast Nurses' Home — Lincoln Institution — Salvation Army Nurses—Glasgow Association . . . . . . . lxi

**The Pen-Bron Hospital** (*concluded*) . . . . . . lxii

**The Prince s of Wales and the Nurses** . . . . . lxiii

**H.R.H. The Princess of Wales** . . . . . . . lxiii

**For Reading to the Sick**—The Good Fight . . . . lxiii

**A Nurse in Natal** (*continued*) . . . . . . . lxiv

**The Nurses' Conversazione** . . . . . . . . lxvi

**Everybody's Opinion.**—Hospitals in Towns—A Welcome Gift for Hospitals—A Neurasthenic Home . . . . lxv

**Examination Questions** . . . . . . . . . lxv

**Notes and Queries** . . . . . . . . . . lxv

**Off Duty**—The Nurses' Bookshelf—A Nurses' Dictionary . lxvi

**The Nurses' Bed** . . . . . . . . . . lxvi

**"Mike"** . . . . . . . . . . . . lxvi

**Where to Go** . . . . . . . . . . . lxvi

**Appointment—Amusements and Relaxation** . . . lxvi

